# ‘Journal of Automatic Chemistry’-the first 10 years

**DOI:** 10.1155/S146392468800001X

**Published:** 1988

**Authors:** P. B. Stockwell


Journal of Automatic Chemistry, Vol. 10, No. 1 (January–March 1988), pp. 1–2

### 'Journal of Automatic Chemistry' – the first 10 years

It only seems like yesterday . . . a familiar saying no doubt, but it is hard to realize that the *Journal* is now entering into its 10th year of operation. During the period we have had a very good and steady supply of papers and we have had very little problem in meeting our publication schedule. At present we have papers available well into our 10th year and with those promised, we should not have any problems in expanding the frequency of publication. Generally speaking, we have been able to present a balanced view of the clinical and industrial scenes, although we have not had many papers submitted relating to process control.

During the last 10 years there have been significant changes in the instrumentation for laboratory automation. When we launched the *Journal* microprocessor technology was receiving much attention and on this aspect I wrote at the time: 'Within the UK, a major interest is centred on microprocessors: this, I feel, distorts the balance of effort in the field of automation. Microprocessors are just tools that the system's designer can use effectively and efficiently if they are required. Microprocessors are certainly no substitute for a proper specification of the analytical problem and the chemistry involved in that automation'. Today, microprocessors are more integrated into instrumentation and not simply additions to the instrument. But the same lessons have to be learnt each time a new concept is introduced. In the area of robotics for example, almost every application simply mimics the manual procedure, rather than addressing the problem from the initial specification stage. Such is the situation that robotic systems are being used where a fraction collector or autosampler would serve the bill, or for procedures like dissolution rate analysis, robotic systems are often preferred to less expensive and equally effective valve switching systems. Jointly, with Glaxo Operations at Barnard Castle, my own company, P S Analytical, has designed and patented a sophisticated building-block for laboratory automation (PSA 40.700 Valve Switching Data Transfer Interface, VCDTI). This selects various valve positions and samples liquid from them into a random sequence. However, this approach is less fashionable than a robotic unit, but is probably the more economic solution. In general, such valve systems have not been as well received as robotics. Figure 1(*a*) shows a schematic representation of the valve switching system designed for dissolution rate analysis.

Computing power has greatly increased and the emergence of inexpensive personal computers with hard disks and up to 30 MByte of storage, as well as colour graphic capabilities, have had a great effect. More and more instrument companies are using PCs to provide control and data processing for sophisticated instruments. The file handling computing that can be used on these is greatly extending the range of tools available to the analyst and chemometrics has emerged as a significant new field of study. The *Journal* has already published some papers on chemometrics and recently two new journals devoted to this subject have been launched. These are welcomed, and should not detract from the general value of *JAC*.

Also, in the first issue of the *Journal* I mentioned the potential of the technique of near infra-red. Recently, with the development of scanning wavelength systems like those available from L T Industries, Pacific Scientific and Guided Wave, the technique has mushroomed and the range of samples has been greatly extended. These units also offer the possibility of analysing samples on a process line directly. Although spectroscopic in nature, this technique offers the analyst the ability to analyse for total solids in milk. These do not have an absorption spectrum on their own in the near infra-red. It can also monitor quantitative physical or sensory sample properties such as 'taste', baking quality or other physical properties of the sample. By being trained to mimic a reference technique, near infra-red can be used to determine the most interesting sample properties such as those affecting the sample's end use. If the sample matrix is consistent then the technique is most valuable, offering speed and simplicity. The on-line capability that it also offers provides another nudge towards the trend to get the analysis out of the laboratory and onto the production line where it can be most quickly applied to solve the problems. Trends can quickly be recognized and corrections made to the process to maximize the company's profitability.

Over the next 10 years, I would expect other analytical developments such as NIR to move more analysis into the plant rather than in the laboratory. However, it should not be forgotten that these techniques, of necessity, require a great deal of effort in the laboratory to check out feasibility and acceptability prior to introduction.

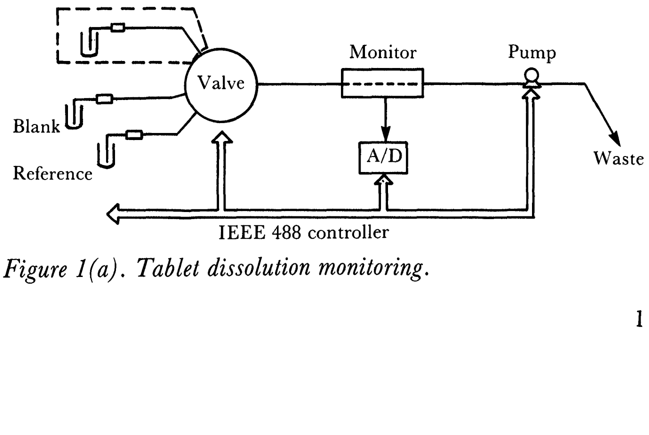

*Figure 1(a). Tablet dissolution monitoring.*

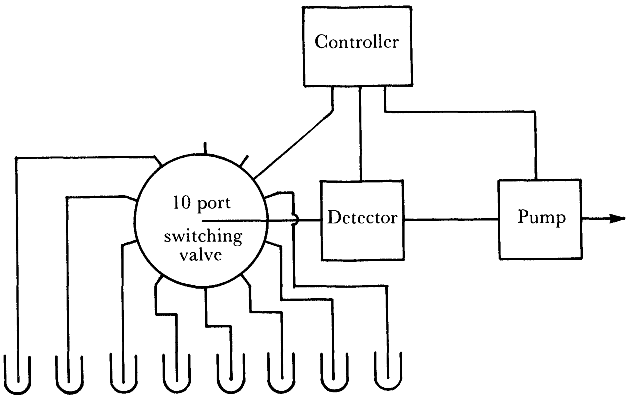

*Figure 1(b). Hydraulic system: narrow bore PTFE tubing to the monitor; low dead volume in-line filters; pumping up to 20 ml/min; 2–3 ml sample size.*

Finally a word of warning – the skill of the analyst is sadly being lost. All too frequently, as an instrument company, one is being asked to provide a solution to analytical problems rather than supplying an instrument. This is a no-win situation, because it is almost impossible for the instrument companies to have detailed experience of the many, many sample types. A great deal of the ability to handle such materials as blood and foodstuffs requires a knowledge of the chemistry involved with the analysis. Hopefully, our teaching establishments will still retain the basic skills of analysis in their courses and will not train a generation of students who can only push buttons on instruments without a clear idea of the basic chemistry involved.

I look forward to receiving many papers on laboratory automation in the next 10 years.

**Peter B. Stockwell**
*Editor*

### FORTHCOMING PAPERS

A computer-controlled potentiometric titrator: *J. D. Stong*

Provisional IFCC guidelines (1987) for listing specifications of laboratory centrifuges: *A. Uldall*

Analytical sonochemistry: a review: *P. Linares et al.*

A systematic evaluation of the Olympus AV5061 as an effective replacement for the SMAC II analyser: *P. B. Hodgin et al.*

Automation of the Beckman liquid scintillation counter for data capture and data-base: *W. Neil et al.*

Automated continuous monitoring of inorganic and total mercury in wastewater and other waters by flow-injection analysis and cold-vapour atomic absorption spectrometry: *S. E. Birnie*

Investigation of the steady-state measurement process: *J. L. Nagy et al.*

### NOTES FOR AUTHORS

*Journal of Automatic Chemistry* covers all aspects of automation and mechanization in analytical, clinical and industrial environments. The *Journal* publishes original research papers; short communications on innovations, techniques and instrumentation, or current research in progress; reports on recent commercial developments; and meeting reports, book reviews and information on forthcoming events. All research papers are refereed.

### Manuscripts

Two copies of articles should be submitted. All articles should be typed in double spacing with ample margins, on one side of the paper only. The following items should be sent: (1) a title-page including a brief and informative title, avoiding the word 'new' and its synonyms; a full list of authors with their affiliations and full addresses; (2) an abstract of about 250 words; (3) the main text; (4) appendices (if any); (5) references; (6) tables, each table on a separate sheet and accompanied by a caption; (7) illustrations (diagrams, drawings and photographs) numbered in a single sequence from 1 upwards and with the author's name on the back of every illustration; captions to illustrations should be typed on a separate sheet. Papers are accepted for publication on condition that they have been submitted only to this *Journal*.

### References

References should be indicated in the text by numbers following the author's name, i.e. Skeggs [6]. In the reference section they should be arranged thus:

to a journal

Manks, D. P., *Journal of Automatic Chemistry*, **3** (1981), 119.

to a book

Malmstadt, H. V., in *Topics in Automatic Chemistry*, Ed. Stockwell, P. B. and Foreman, J. K. (Horwood, Chichester, 1978), p. 68.

### Illustrations

Original copies of diagrams and drawings should be supplied, and should be drawn to be suitable for reduction to the page or column width of the *Journal*, i.e. to 85 mm or 179 mm, with special attention to lettering size. Photographs may be sent as glossy prints or as negatives.

### Proofs and offprints

The principal or corresponding author will be sent proofs for checking and will receive 50 offprints free of charge. Additional offprints may be ordered on a form which accompanies the proofs.

*Manuscripts should be sent to either Dr P. B. Stockwell or Ms M. R. Stewart, see inside front cover.*

